# Identification of Anchor Genes during Kidney Development Defines Ontological Relationships, Molecular Subcompartments and Regulatory Pathways

**DOI:** 10.1371/journal.pone.0017286

**Published:** 2011-02-28

**Authors:** Rathi D. Thiagarajan, Kylie M. Georgas, Bree A. Rumballe, Emmanuelle Lesieur, Han Sheng Chiu, Darrin Taylor, Dave T. P. Tang, Sean M. Grimmond, Melissa H. Little

**Affiliations:** Institute for Molecular Bioscience, The University of Queensland, St. Lucia, Australia; University of Southern California, United States of America

## Abstract

The development of the mammalian kidney is well conserved from mouse to man. Despite considerable temporal and spatial data on gene expression in mammalian kidney development, primarily in rodent species, there is a paucity of genes whose expression is absolutely specific to a given anatomical compartment and/or developmental stage, defined here as ‘anchor’ genes. We previously generated an atlas of gene expression in the developing mouse kidney using microarray analysis of anatomical compartments collected via laser capture microdissection. Here, this data is further analysed to identify anchor genes via stringent bioinformatic filtering followed by high resolution section *in situ* hybridisation performed on 200 transcripts selected as specific to one of 11 anatomical compartments within the midgestation mouse kidney. A total of 37 anchor genes were identified across 6 compartments with the early proximal tubule being the compartment richest in anchor genes. Analysis of minimal and evolutionarily conserved promoter regions of this set of 25 anchor genes identified enrichment of transcription factor binding sites for *Hnf4a* and *Hnf1b*, *RbpJ* (Notch signalling), PPARγ:RxRA and COUP-TF family transcription factors. This was reinforced by GO analyses which also identified these anchor genes as targets in processes including epithelial proliferation and proximal tubular function. As well as defining anchor genes, this large scale validation of gene expression identified a further 92 compartment-enriched genes able to subcompartmentalise key processes during murine renal organogenesis spatially or ontologically. This included a cohort of 13 ureteric epithelial genes revealing previously unappreciated compartmentalisation of the collecting duct system and a series of early tubule genes suggesting that segmentation into proximal tubule, loop of Henle and distal tubule does not occur until the onset of glomerular vascularisation. Overall, this study serves to illuminate previously ill-defined stages of patterning and will enable further refinement of the lineage relationships within mammalian kidney development.

## Introduction

Many diseases of the kidney stem from disruptions to the transcriptional programs involved in normal kidney development [1]. Such disruptions, resulting from both genetic and environmental factors, can affect overall renal function in postnatal life. Indeed, predisposition to renal disease in humans is inversely related to the number of functional epithelial nephrons per kidney [2], a parameter completely determined during kidney development. Understanding this molecular pathogenesis has been a major aim of kidney organogenesis research [1]. To do this, it is critical to understand the extent and origins of cellular complexity within the developing kidney.

Kidney organogenesis in mammalian species is highly conserved anatomically and molecularly. Hence, the field has made considerable use of model organisms, predominantly mouse and rat, to study the process. The mammalian kidney is a complex organ containing more than 25 distinct functional cell types [3], [4]. These arise from one of two intermediate mesoderm-derived cell populations, the metanephric mesenchyme (MM) and the ureteric bud [5]. The UB forms a dichotomously branching epithelial tree, giving rise to the cell types that make up the collecting ducts of the kidney and the ureter that connects the kidney with the bladder. This involves considerable regional specification to ensure the vast variation in water permeability between the water-reclaiming collecting ducts and the water impermeable conduit that is the ureter. Regionalisation within this compartment is also critical for nephron formation as key proteins expressed by the tips of this epithelium signal to the surrounding mesenchyme to initiate nephrogenesis. Equally, there is now evidence that the segmentation and patterning of the nephrons themselves is dictated via the secretion of distinct Wnt proteins from the collecting duct tree [6]. The MM gives rise to the cap mesenchyme (CM), which in turn forms all of the tubular elements of the nephron other than the collecting ducts via a process of mesenchyme to epithelial transition (MET) immediately adjacent to the tip of the advancing UB branches [7], [8], [9]. As a result, the human kidney forms up to 2 million nephrons per kidney. An individual mature nephron is comprised of at least 14 distinct functional segments [10], [11], each of which contains cell types that play specific roles in water and solute retention and loss. While patterning and segmentation must occur to reach this level of specialization, only the earliest events have been well studied. Hence the role of *Wnt9b* in the initiation of MET and the subsequent requirement for *Wnt4* expression to proceed through this event is well documented [12], [13], as is the requirement for *Notch2* for proximal tubule specification [14]. The regulation of subsequent events remains to be dissected. The remainder of the MM does not undergo MET, instead giving rise to elements of the interstitium of the kidney. The interstitium as a whole is highly heterogeneous, containing fibroblasts, resident macrophages, vascular, perivascular/smooth muscle, lymphatic and neural tissue of the kidney [15], [16]. While some interstitial cell types are likely to migrate into the interstitium during development, including the resident macrophages/dendritic cells [17], the origin and lineage relationships of these components is still unresolved, as are their roles during normal kidney homeostasis and in response to renal injury [18].

The development of conditional transgenic strains in the mouse has particularly assisted in defining our existing understanding of ontological relationships during kidney development. For example, the *Six2* gene is regionally-enriched in the CM during kidney development [19]. Using a Six2GFPCre transgenic line, Kobayashi et al (2008) demonstrated that all epithelial portions of the nephron apart from the collecting duct were derived from CM [7]. Using the same transgenic line, it was shown that during renal injury no interstitial stem cells could contribute to tubular repair as there is no apparent dilution of the nephron epithelium with cells that did not originally express *Six2* [20]. Finally, Georgas et al (2009) showed that the connecting segment cells at the point of fusion between the collecting duct tips and the nephron tubules are derived from the CM and not the UB [9]. The power of these analyses rests with the compartment-specific expression of the *Six2* gene. The identification of additional compartment-specific genes is needed to advance our understanding of kidney development, notably during the later stages of patterning, segmentation and differentiation. The obvious solution to this would appear to be gene expression analysis of kidney development.

While global analysis of gene expression in the entire developing kidney has been of limited value given the cellular complexity of this organ [21], [22], [23], [24], we have previously contributed to the most comprehensive compartmental analysis of any developing organ, in which Affymetrix microarray expression profiling was performed on 15 distinct temporospatial anatomical compartments of the developing mouse kidney collected via laser capture micro-dissection or FACS [25]. This allowed for the identification of genes and gene networks enriched during different processes of kidney development, but also highlighted the paucity of genes absolutely restricted in expression to a single compartment in time and space.

Microarray compartments in Brunskill et al (2008) [25] were based primarily upon identifiable anatomical and/or regional subdivisions rather than established molecular or ontological entities. Other gene expression studies during organogenesis [26], [27], [28] have demonstrated the need for complimentary high resolution validation to more finely dissect the relationship between gene expression and anatomical organization. It was anticipated that this would reveal additional ‘molecularly-defined’ compartments more representative of key developmental processes, including segmentation and patterning, and possibly also identify specific cell types within complex and heterogeneous compartments. In the study presented here, we have extended the analysis of the kidney development gene atlas using high resolution section *in situ* hybridisation (SISH). A bioinformatic method was devised for the stringent prediction of ‘anchor’ genes, defined as a gene whose expression was restricted to one temporospatial anatomical compartment. A total of 200 genes across 11 anatomical compartments were analysed with 46% of genes (92 genes) being compartment-enriched and an additional 18.5% (37 genes) representing anchor genes for a defined temporospatial structure (overall validation of bioinformatic selection of 64.5%). As such, these anchor genes will fuel the generation of further mouse resources for lineage tracing, thereby extending our understanding of kidney organogenesis and ultimately molecular pathogenesis within the kidney. Equally, compartment-enriched genes redefined the developing kidney atlas into molecular events rather than regional or anatomical structures, identifying markers able to subdivide the processes of nephron segmentation, collecting duct functionalisation and interstitial differentiation.

## Results and Discussion

### Selecting potential anchor genes via reanalysis of the atlas of kidney development

**Figure 1:**
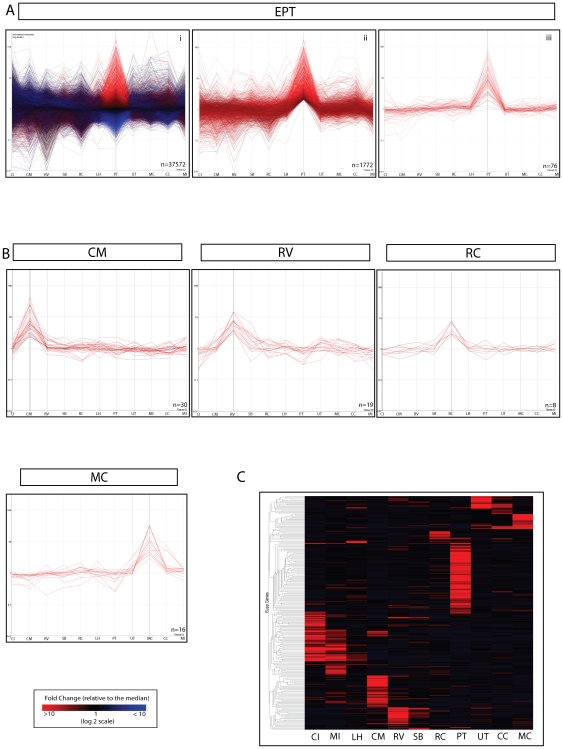
Identification of candidate anchor genes. Microarray data generated by Brunskill et al (2008) was analysed to identify potential compartment specific gene expression in eleven kidney compartments - Cortical interstitium (CI), medullary interstitium (MI), loop of Henle (LH), cap mesenchyme (CM), renal vesicle (RV), S-shaped body [46], renal corpuscle (RC), early proximal tubule (PT), ureteric tip (UT), cortical collecting duct [15] and medullary collecting duct (MC). **A**) The preliminary selection for candidate anchor genes/markers used for all compartments is exemplified using EPT. i) Identification of differentially expressed genes across all profiled compartments (ANOVA p<0.01) with EPT genes highlighted in red; ii) Genes up-regulated within the compartment of interest were selected based on normalized values (fold-change) >2 (log scale) against the median. iii) Final candidate genes for EPT; Genes were further filtered for absolute restricted expression by excluding probesets that were expressed at ≥2fold in other subcompartments, then ranked on median signal intensity values (<200RFU (raw fluorescent units)) and raw signal intensity values (>100RFU) across all compartments. **B**) Expression profiles of compartment specific genes selected from CM, RV, RC and MC for validation via SISH. **C**) Global view of distribution of expression for all 11 compartments analysed.

Brunskill et al (2008) [25] reported Affymetrix expression profiling for 15 compartments, 11 of which were isolated from embryonic day (E) 15.5 developing murine kidney. Microarray data from these 11 anatomical compartments represented interstitial/mesenchymal elements (cap mesenchyme, CM; medullary interstitium, MI; cortical interstitium, CI), anatomical subdivisions of the ureteric epithelium (ureteric tip, UT; cortical collecting duct, CCD; medullary collecting duct, MCD) and CM-derived elements of the nephron (renal vesicle/Stage I nephron, RV; S-shaped body/Stage II nephron, SSB; early proximal tubule, EPT; Loop of Henle, LH and renal corpuscle, RC). A list of putative anchor genes were selected as those displaying; a) the most significant differential expression ratio (>2 x median expression fold change), b) high raw signal intensity (>100 RFU) above background and c) low median signal intensity in all other subcompartments examined (Figure 1). As can be seen in Figure 1, there was considerable variation in the median expression fold change and number of putative anchor genes identified between compartments. Early proximal tubule showed the largest number of highly differentially expressed genes whereas compartments representing transient developmental stages (RV, SSB) or probable cellular heterogeneity (MI, CI) showed very few selected genes. As a lack of anchor genes may also have arisen due to a lack of association between the physical compartment selected and any molecular association or due to contamination of samples during isolation, a parallel comparison of a subset of 7 compartments (UT, RC, MCD, RV, EPT, MI, and SSB) was also performed. A complete list of the 251 genes prioritised for validation from both comparisons is listed in Table S1.

### Investigating apparent false negatives at the level of microarray

Validation via *in situ* hybridisation only proceeded for those genes selected according to the criteria determined for selection of potential anchor genes. The approach taken here is distinct from our previous analysis [25] and hence not all genes identified as enriched were included for validation. For example, *Prnp*, a gene we have shown to mark a distinct subcompartment of the SSB, was not selected as it is also expressed at a significant level in RV. As this approach did not identify a number of genes commonly regarded as marking specific renal compartments, a retrospective analysis was performed to determine why such genes were excluded. Four major issues arose. 1) Insufficient differential in expression, e.g. *Wnt11* and *Wnt7b*, regarded as specific to the ureteric tip and collecting duct respectively, were not selected due to insufficient differential expression between this and all other compartments. 2) Absence from microarray probeset, e.g. *Crlf* (syn: *CLF-1*), previously reported as specific to the UT [29], was not represented on the Affymetrix microarrays. 3) Evidence from microarray for an expression pattern contrary to that previously reported, e.g. *Pf4* (*Cxcl4*), previously reported as a UT marker [29],[30], was clearly present in the EPT compartment. 4) Genes thought to mark a specific compartment were also expressed in other regions, e.g. *Cadherin 16* (*Cdh16*), although previously described as a marker of collecting duct [31], was also expressed by microarray in the EPT. On occasion this may reflect the accuracy of the selection of material for microarray, resulting in false negatives. For example, *Six2*, a well known marker of the CM was also detected in the MI compartment. While this may reflect contamination of the MI compartment with CM, there was no such contamination detected in the more closely located CI.

### Validation of anchor genes using high-resolution mRNA *in situ* hybridisation

Of the 251 putative anchor genes selected, 200 were analysed using high resolution section *in situ* hybridisation (SISH) of paraffin-embedded E15.5 murine kidney. A subset of genes was also examined in adult kidney. In all cases, expression patterns were annotated in accordance with the anatomical ontology previously generated for the mouse urogenital system [4]. Each gene was then classified as *specific* (expression only present in a single compartment; validated anchor gene), *enriched* (expression present in the correct compartment and at least one other compartment; marker gene), *ubiquitous* (ubiquitous expression in all compartments), *not-detected* (no detectable expression) or *non-specific* (expression detected in an unexpected compartment). As microarray data was not available for all kidney compartments, some genes validated with respect to the other ten compartments but were not regarded as anchor genes as they were found to also be expressed in a compartment not included in the analysis (e.g. distal tubule). The fully annotated expression patterns, riboprobe details and all ISH images for the complete set of validated genes are available on the GUDMAP website (http://www.gudmap.org).

**Figure 2:**
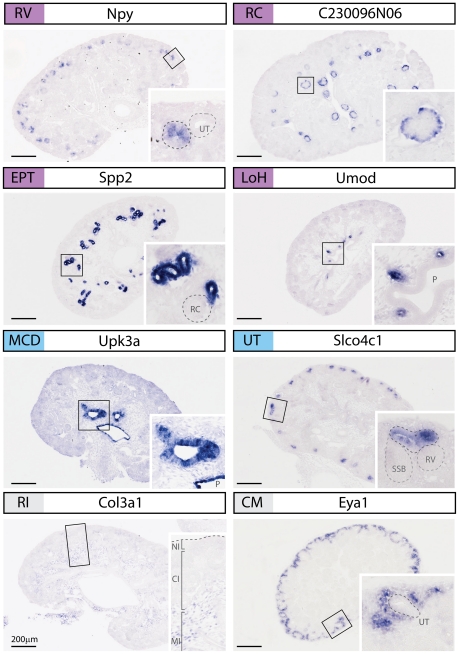
High resolution SISH validating anchor genes from a variety of developmental compartments within the developing kidney. Representative SISH images of the 15.5dpc kidney are shown for anchor genes identified for the renal vesicle (RV), renal corpuscle (RC), early proximal tubule (EPT), loop of Henle (LoH), medullary collecting duct (MCD) and ureteric tip (UT). Marker genes for the renal interstitium (RI) and cap mesenchyme (CM) are also shown. Col3a1 is expressed in the cortical (CI), medullary (MI) and perihilar interstitial compartments but absent from the nephrogenic interstitium (NI). Eya1 is expressed in the CM and a subset of the adjacent NI. The location of the high magnification inset images is outlined. All scalebars are 200 mm, P = pelvis, SSB = S-shaped body.

The percentage validation (combining specific and enriched) for all compartments was 64.5%, however this did vary considerably between compartments (Table S1) and anchor genes were not detected for all compartments. No true anchor genes were detected for the CM due to the very low signal for many of the selected genes (e.g. *Rspo3*, *Disp1*, *Cacnb4*, *Adcy8*). Other genes (*Eya1*; Figure 2) could be detected in surrounding interstitium either between early nephrons and/or in pretubular aggregate as well as the CM, in contrast to the previous literature [32]. The lack of an SSB anchor gene is likely to reflect the fact that this structure is a continuum of nephron development with no unique gene expression or that it was difficult to definitively identify this stage from others at isolation based upon PNA staining. Very few LH anchor genes were identified and this gene set may have included surrounding interstitium based upon the overlap between array signals between these compartments.

### Investigating false positives at the level of microarray and riboprobe design: gene-centric vs. transcript-centric riboprobes

The success of the validations depended on the ability to replicate the gene expression detected in the microarray study. While the majority of the genes that were validated produced an expected signal pattern (129/200), 71 other gene probes yielded false positive signal (non-specific, not detected, or ubiquitous). Sources of these variations are mostly likely due to the technical challenges from comparing microarrays and ISH. These variations include contamination of LCM samples from spatially adjacent compartments during sampling [25] as discussed above. A key issue was selecting an ideal dynamic range of signal detection and specificity during the microarray analysis. While the range of raw microarray signals successfully validated extended from 100 to 10,000 RFU, less than 50% of probes displaying microarray expression levels of <500 RFU could be validated by SISH of paraffin embedded material, highlighting the limits of SISH signal detection [25]. Also, different cell-densities are likely to influence the mean signal intensities of each subcompartment and therefore, a generalized threshold of detection across all samples does not reflect the individual dynamic range.

Another source of variation comes from discrepancies of microarray and SISH probes representing a gene. One of the issues lies in the sequence length of the microarray probeset and SISH riboprobe. The riboprobes used in this study were typically designed within the 500–800 bp range, while the Affymetrix probes are 25 mers. The shorter probes are more likely to target specific transcripts whereas the longer probes can span across several transcripts and are therefore more likely to pick up different signal combinations picked up by each transcript [33]. This suggests that riboprobes that directly overlap Affymetrix probes along the target transcript, ‘transcript-centric’ probes, were more likely produce replicable signal during SISH validation than gene-centric designs. We reviewed the riboprobe localization along the locus relative to the Affymetrix probeset for riboprobes showing *non-specific* expression, as these probes are capable of providing signal (i.e. no ubiquitous background signal). *Non-specific* riboprobes showed less than 80% overlap with the Affymetrix probeset which may be a contributing factor towards the inaccuracy of the riboprobe and could be avoided during riboprobe design.

Since microarray and SISH based comparisons are common for the validation of gene targets, a riboprobe design pipeline that minimizes discrepancies in expression validation arising from transcriptional complexity was created by consolidating the Affymetrix probeset locations into the design of SISH riboprobes. Each of the eleven 25 mer oligo-probes in a given Affymetrix probeset were mapped against a non-redundant set of mouse cDNA transcripts (FANTOM3 cDNA clones) to obtain overlapping regions to serve as sequence templates for riboprobe primer design via the Primer3 program [34]. The overall popularity of the Affymetrix Mouse Genome 430.2 platform across many murine studies (over 1400 experimental series in GEO, (GPL: 1261)) and within the GUDMAP consortium [35], lead to the creation of a riboprobe primer design tool (http://uqgudmap.imb.uq.edu.au/riboprobe_design/). Here, each probeset entered, results in the generation by Primer3 of the corresponding riboprobe primer sequences. This then links to the complete set of primer statistics in the Sequence Manipulation Suite (http://www.bioinformatics.org/sms2/) and the In-Silico PCR program from the UCSC Genome Browser [36] which predicts the specificity of the amplified riboprobe region.

### Definition of molecular compartments of kidney development across time, developmental process and space

**Figure 3:**
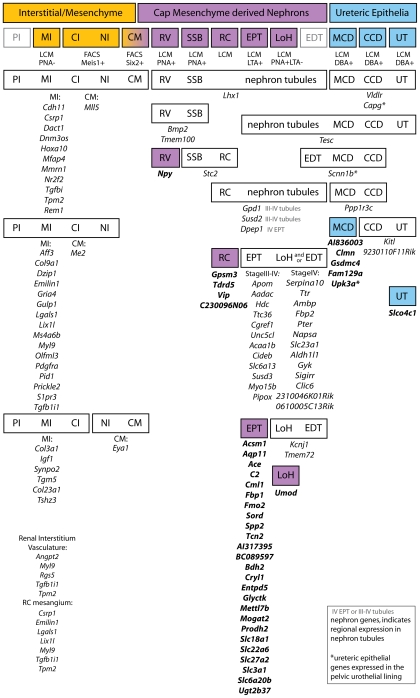
Correlation between anatomical compartments used for microarray analysis with molecular compartments revealed via SISH. The 3 major tissue compartments of the kidney are represented by the top row of painted bars (yellow, purple, blue). Below this, the second row indicates the anatomical subdivision of these into 14 compartments. The 11 compartments profiled by microarray are painted and the method used to isolate each is indicated below the bar [25]. PI and EDT were not isolated for microarray, and CI and NI were isolated as one compartment. The bars in the rows below these indicate the regional gene expression patterns observed by high resolution SISH. The expression patterns are shown from broadest (top) to the most restricted (bottom) and the genes observed with each of these patterns are listed below the bar. The painted gene expression bars (yellow, purple, blue) indicate restricted expression in only one compartment and the genes listed below each of these are the anchor genes (bold type). PI, MI, CI and NI = perihilar, renal medullary, renal cortical and nephrogenic interstitium; CM = cap mesenchyme; RV = renal vesicle; SSB = S-shaped body; RC = renal corpuscle (StageIII-IV); EPT = early proximal tubule; LoH = immature and anlage of loop of Henle; EDT = early distal tubule; MCD and CCD = medullary and cortical collecting duct; UT = ureteric tip. Below each of the interstitial/mesenchyme expression bars, MI: and CI: indicate the microarrary compartment used to identify the genes. Some genes identified in the renal interstitium were also expressed in the vasculature of the kidney and/or the mesangial tissue of renal corpuscles and these genes are listed (bottom, left).

In total, 37 anchor genes were identified representing 6 compartments within the developing kidney (Table 1, Figure 2, Figure 3). Four anchor genes for the renal corpuscle were identified, three of which were restricted to the visceral epithelium/podocytes (*Gpsm3*, *Tdrd5*, *C230096N06*) and one marking the juxtaglomerular arterioles (*Vip*). *Gpsm3* and *C230096N06* were expressed by both Stage III and IV nephrons however *Vip* and *Tdrd5* were restricted to the more mature Stage IV RC. A single LOH anchor gene, *Umod*, was identified. This is a well known marker of the LOH and mutations in *Umod* have been associated with a range of chronic kidney diseases, such as hyperuremic nephropathy (OMIM: 162000) and medullary cystic kidney disease (OMIM: 603860). While many genes are expressed in the renal vesicle, as previously reported [9], only a single gene (*Npy*) showed expression restricted to this earliest stage of the nephron. Anchor genes for EPT, MCD, UT are discussed later.

**Table 1:** Final list of anchor genes determined via SISH listing their spatial location and any known association with renal disease.

Affymetrix ID	Raw	Fold-change	Median	Gene Symbol	Compartment	GUDMAP ID
1427034_at	916.2	12.3	74.5	*Ace*	EPT	GUDMAP:13511
1424758_s_at	1862.1	38.3	48.6	*Acsm1*	EPT	GUDMAP:10782
1451760_s_at	501.6	13	39.9	*AI317395*	EPT	GUDMAP:9158
1429254_at	708.2	14	49.9	*Aqp11*	EPT	GUDMAP:13930
1451681_at	692.0	22.2	31.1	*BC089597*	EPT	GUDMAP:13513
1453011_at	794.5	11	75.0	*Bdh2*	EPT	GUDMAP:9152
1416051_at	897.8	15.7	57.1	*C2*	EPT	GUDMAP:9118
1418013_at	7504.8	45	168.1	*Cml1*	EPT	GUDMAP:9133
1447112_s_at	2542.7	10	243.0	*Cryl1*	EPT	GUDMAP:11171
1417382_at	4464.5	13	344.0	*Entpd5*	EPT	GUDMAP:9175
1448470_at	3072.5	110	28.0	*Fbp1*	EPT	GUDMAP:9177
1435459_at	313.3	15	20.3	*Fmo2*	EPT	GUDMAP:9128
1424995_at	278.4	12.5	22.3	*Glyctk*	EPT	GUDMAP:13504
1416980_at	925.0	21	44.8	*Mettl7b*	EPT	GUDMAP:9161
1455099_at	1374.9	13.7	100.6	*Mogat2*	EPT	GUDMAP:13580
1432099_a_at	599.4	11	52.9	*Prodh2*	EPT	GUDMAP:9124
1426595_at	3224.5	24	131.7	*Slc18a1*	EPT	GUDMAP:9039
1417072_at	2460.4	37.1	66.4	*Slc22a6*	EPT	GUDMAP:9116
1416316_at	10454.9	118	88.3	*Slc27a2*	EPT	GUDMAP:9179
1448741_at	4187.5	95.4	43.9	*Slc3a1*	EPT	GUDMAP:9141
1422899_at	1178.6	35.8	32.9	*Slc6a20b*	EPT	GUDMAP:9036
1438183_x_at	834.2	18.5	45.2	*Sord*	EPT	GUDMAP:9136
1418916_at	10339.9	192.9	53.6	*Spp2*	EPT	GUDMAP:9147
1447800_x_at	12193.4	11	1129.0	*Tcn2*	EPT	GUDMAP:9178
1449890_at	1991.4	179	11.2	*Ugt2b37*	EPT	GUDMAP:9180
1426252_a_at	323.9	27.3	11.9	*Umod*	LoH	GUDMAP:9104
1449104_at	1387.7	31.6	43.9	*Upk3a*	MCD	GUDMAP:9904
1422567_at	720.7	14	51.9	*Fam129a*	MCD	GUDMAP:11019
1430641_at	2080.5	29.9	69.6	*Gsdmc4*	MCD	GUDMAP:11309
1439117_at	230.7	4.3	53.5	*Clmn*	MCD	GUDMAP:13648
1436099_at	328.9	3.2	101.2	*AI836003*	MCD	GUDMAP:13573
1456391_at	356.6	7.8	45.5	*Tdrd5*	RC	GUDMAP:9106
1418396_at	328.7	3.1	105.4	*Gpsm3*	RC	GUDMAP:13584
1446524_at	309.7	13.3	23.2	*C230096N06*	RC	GUDMAP:13516
1428664_at	193.0	8.9	21.6	*Vip*	RC	GUDMAP:13795
1419127_at	740.6	6.8	108.5	*Npy*	RV	GUDMAP:8964
1460616_at	1042.8	66.0	15.8	*Slco4c1*	UT	GUDMAP:11316

Affymetrix IDs represent probesets from the Affymetrix 430.2 Mouse platform (GEO database ID: GPL 1261) with corresponding raw signal intensity values (Raw) measured in raw fluorescent units (RFU), normalized raw signal intensity in log scale 10 (Fold-change) within compartment of interest (Compartment) and median raw signal intensity of probeset against other subcompartments (Median); SISH images available on the GUDMAP website (http://www.gudmap.org) via corresponding Accession IDs (GUDMAP ID). Compartment: EPT  =  early proximal tubule; LoH  =  loop of Henle; MCD  =  medullary collecting duct; RC  =  renal corpuscle; RV  =  renal vesicle; UT  =  ureteric tip.

In addition, a further 77 genes showed specificity for previously undefined temporal or spatial compartments. This enabled the redefinition of initially anatomical subdivisions into molecular compartments more closely reflecting the biology of kidney development. Figure 3 illustrates the relationship between the initial compartments collected for microarray, indicating the basis upon which they were collected. Initial compartments represented three developmental processes; interstitial differentiation, nephron induction and segmentation and ureteric epithelial functionalisation. A number of these genes have already been associated with postnatal renal disease or congenital anomalies of the kidney. Mutations in *Kcnj1* are associated with Bartter syndrome (OMIM: 241200), a life-threatening disorder including multiple developmental abnormalities.

Despite validation of a large number of genes, no gene was identified as an anchor gene for either the MI or CI. Instead, the pattern of gene expression observed within the interstitium reflected the fact that this compartment contains a variety of cell types that are present throughout all regions of the interstititum. Eleven interstitial genes were present in all mesenchymal interstitium including the CM, 16 genes were present in all interstitial regions other than the CM and 6 genes were present in MI and CI but not nephrogenic insterstitium, perihilar interstitium or CM. It should be noted that the CI compartment was isolated using FACS sorting of *Meis1*+ GFP mice, a gene whose expression does extend into the CM (see GUDMAP data Accession ID GUDMAP:12521). However, overall the absence of markers that distinguish individual interstitial compartments also supports the notion of commonality in cellular composition within much of the interstitial space. It also highlights the need for ultimate compartmentalization of this organ down to the cellular level.

### Segmentation of the ureteric compartment

**Figure 4:**
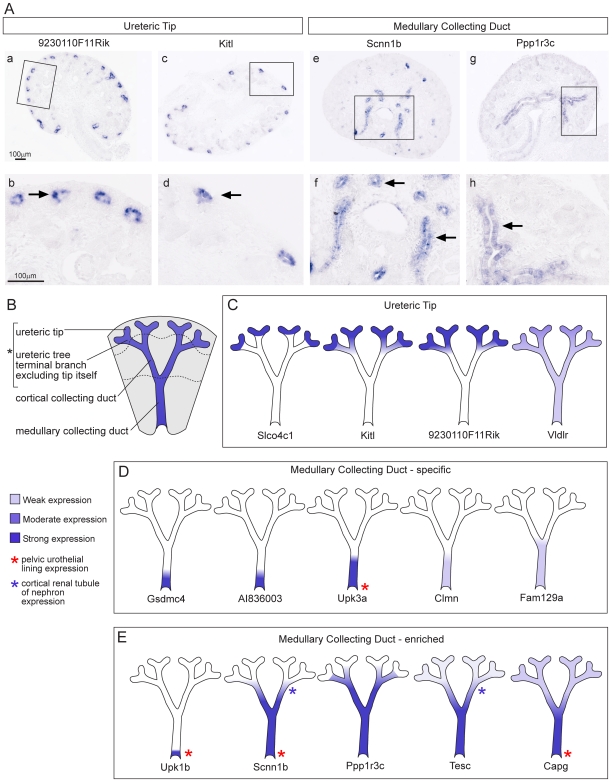
Expression of marker genes in the ureteric tree at 15.5dpc. Three compartments were analysed for anchor genes in the ureteric tree; the ureteric tip including the uretric tree terminal branch (UT), the cortical collecting duct (CCD) and medullary collecting duct (MCD). **A**) Examples of expression patterns seen by SISH are shown for UT and MCD genes. The top panel shows kidney sections with the region enlarged shown below. Arrows in b,d  =  UT and f,h  =  MCD expression. The genes identified as potentially CCD-specific did not validate by SISH. SISH images and text-annotated expression patterns for all genes are available on the GUDMAP website (http://www.gudmap.org). **B**) Schematic of 15.5dpc kidney divided into nephrogenic zone (top), renal cortex, and medulla showing segmentation of the ureteric tree. *indicates the regions collected by LCM representing the UT compartment. The expression of each gene giving either a specific or enriched expression pattern was painted onto the schematic and is shown in **C**) UT genes **D**) MCD specific anchor genes and **E**) MCD enriched genes. *Slco4c1* was identified as the only UT-specific anchor gene.

While it is known that specific factors secreted from the tips of the ureteric tree are critical for the induction of nephron formation, the specification, segmentation and differentiation of non-tip regions of the ureteric epithelium is not well understood. Many well known ureteric tree markers are ubiquitously expressed throughout the ureteric epithelia during development, including *Calb1* and *Hoxb7* [37], [38]. While previous studies have sought differential markers of ureteric trunk versus tip [21], [29], [30], few have been well defined other than *Wnt11* in the tip [39]. Even the widely reported tip marker, *Ret*, while enriched in the ureteric tip, is also expressed weakly in the ureteric trunk [39]. Three spatially defined compartments were isolated from the ureteric epithelium in the initial microarray analysis; UT, CCD and MCD. Validation of predicted anchor genes within these subcompartments identified *Slco4c1* as a novel UT anchor gene, five MCD anchor genes (*Gsdmc4*, *Clmn*, *AI83600*, *Fam129a*, *and Upk3a*) but no CCD anchor genes. However, a cohort of seven additional genes revealed a complex set of overlapping gene expression patterns indicating a much greater level of segmentation of this epithelial compartment (Figure 4). Two genes, *Tesc* and *Scnn1b* were expressed in the ureteric epithelia as well as the adjacent distal nephron tubules. Genes specific to or enriched in MCD included genes involved in epithelial differentiation and specialisation. *Gsdmc4*, part of the Gsdmc gene cluster, also shows epithelial-specific expression in the skin and gut [40]. Mutations in *Upk3a*, which encodes a protein critical to urothelial plaque formation, can result in renal adysplasia including vesicoureteral reflux and echogenic cystic kidneys [41]. This set of markers can be used to further understand functional differentiation along the ureteric tree.

### Distinct temporospatial expression domains within the developing proximal tubule

**Figure 5:**
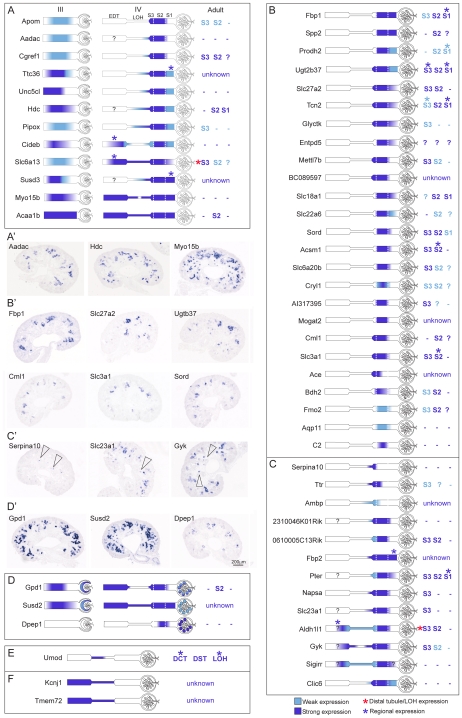
Identification of distinct ontological markers of renal tubule segmentation and patterning. For the early proximal tubule (EPT) and loop of Henle (LOH) compartments of the nephron, expression patterns determined by SISH at 15.5dpc were painted onto a schematic of stage III and IV nephrons (see Figure S1). Both tubular and renal corpuscle (RC) structures were painted and expression strength is indicated by colour. Expression in the adult nephron is indicated in text on the right of each schematic (renal proximal tubule S1 S2 S3 segments, DCT distal convoluted tubule, DST distal straight tubule and LOH) and – indicates expression is absent. Unknown  =  expression was not examined?  =  uncertain expression. Red* indicates that these EPT genes also showed expression in a subset of the distal tubules in the adult kidney, *Slc6a13* (DST) and *Aldh11* (DCT, DST). EPT genes with restricted expression in the nephron tubules were divided into 3 categories; (**A**) early genes showing expression in Stage III and IV tubules, (**B**) late genes restricted to EPT of Stage IV tubules, the EPT anchor genes and (**C**) late genes with expression in EPT and other tubular segments (immature loop of Henle and/or early distal tubule). (**D**) EPT genes which showed additional expression in the renal corpuscle nephron compartment represent an additional category of enriched EPT genes. (**A′–D′**) Expression patterns in the kidney at 15.5dpc by SISH are shown for representative genes from each of the EPT spatiotemporal categories. In C′, arrowheads indicate expression in immature LOH. For the LOH compartment, three genes were identified (**E**) *Umod* a LOH-specific anchor gene and (**F**) *Kcnj1* and *Tmem72* LOH markers also expressed in the early distal tubule (EDT). SISH images and text-annotated expression patterns for all genes are available on the GUDMAP website (http://www.gudmap.org).

The strength of the anchor gene analysis was especially demonstrated within the EPT compartment in which 25 anchor genes were identified (see Table 1, Figure 3). The EPT genes demonstrated different regional expression patterns across the tubular segments of the nephron, with regional patterns observed within the Stage IV EPT itself, which could be further subdivided into presumptive S1, S2 and S3 segments (see Figure S1). Figure 5 shows representations of the pattern of expression of each of these EPT anchor genes in the Stage IV nephron. The majority of EPT anchor genes maintained this specific pattern of expression in the proximal tubule of the adult kidney, suggesting very early specification of this tubular segment (see Figure 5 B, Figure S2). As well as identifying many EPT anchor genes, a further 34 genes (identified within the RV, SSB, RC, EPT and LOH compartments) allowed the subdivision of cap mesenchyme derived nephron development into distinct temporospatial gene expression patterns including early (*Lhx1*) and later markers of pan-nephron development (*Gpd1*, *Susd2*, *Dpep1*), early nephron (*Bmp2*, *Tmem100*), presumptive podocyte (*Stc2*) and LOH/distal tubule (*Kcnj1*, *Tmem72*) (Figure 3). One group of 12 genes displayed expression in the tubules of Stage III nephrons. All genes in this group were also expressed in Stage IV nephrons (Figure 3, Figure 5A) but were not restricted to the proximal tubule. Other genes do not commence expression until the differentiation of the nephron into Stage IV (maturing nephron). This included the 25 anchor genes whose expression was restricted to the proximal tubules (Figure 3, Figure 5B) together with a further 13 genes whose expression extended beyond the proximal tubule into other tubular segments (immature loop of Henle and/or early distal tubule) (Figure 3, Figure 5C). As with EPT anchor genes, many of these marker genes maintained the same regional domains of expression in the adult proximal tubule (Figure 5, Figure S2). This suggests that segmentation of the early nephron into proximal, distal and LOH elements does not occur until the formation of a Stage IV nephron.

### Transcriptional regulation and gene ontological relationships of proximal tubule anchor genes

The large number of anchor genes identified in the proximal tubule provided the basis to perform downstream analyses to understand transcriptional control of tissue-specific expression. The proximal promoters of EPT anchor genes were used to identify over-represented transcription factor binding motifs defined at −500/+200 of RefSeq annotated transcription start sites (referred to as “Core”) using the CLOVER algorithm [42] against the JASPAR motif library [43](see Methods). A second set of sequences containing only evolutionarily conserved regions within the promoter was also used in a separate analysis (referred to as “ECR”). The collective results are summarized in Table 2. In total, 10 TF motifs were statistically enriched in EPT anchor gene promoters using both Core and ECR analyses. Two of these transcription factors *RbpJ* and *Hnf1a* have been previously shown to directly affect proximal tubule development and function [14], [44]. *Hnf1b* and *Hnf4a* transcription factors are associated with kidney development [45], [46], [47], [48], [49] however no direct involvement during proximal tubule development has been reported to date. Almost all EPT anchor gene promoters (22/25) showed binding site for *Hnf4a*. SISH analysis of *Hnf1b* and *Hnf4a* shows expression within the proximal tubule and other nephron segments with *Hnf1b* showing collecting duct expression and *Hnf1a* showing S-shaped body expression (see GUDMAP data Accession IDs GUDMAP:12560 and GUDMAP:12531 respectively).

**Table 2:** List of predicted transcription factor binding site motifs within proximal tubule anchor gene set promoters.

TF Motif	TF class	Prediction	# genes	p-value	Target Genes	Renal defect	Expression in developing kidney	EPT expression
Hnf4a	Nuclear Receptor	Core	22/25	0	*Spp2*, *Prodh2*, *Aqp11*, *Slc18a1*, *Glyctk*, *Slc6a20b*, *Mettl7b*, *AI317395*, *BC089597*, *Tcn2*, *Ace*, *Entpd5*, *Cryl1*, *Slc3a1*, *C2*, *Slc22a6*, *Fmo2*, *Slc27a2*, *Bdh2*, *Ugt2b37*, *Acsm1*, *Mogat2*	Y	early tubule	Y
PPAR::RXRA	Nuclear Receptor, heterodimer	Core, ECR	18/25	0.001-0.017	*Spp2*, *Sord*, *Prodh2*, *Slc18a1*, *Glyctk*, *Mettl7b*, *BC089597*, *Ace*, *Fbp1*, *Cryl1*, *Slc3a1*, *C2*, *Slc22a6*, *Fmo2*, *Slc27a2*, *Ugt2b37*, *Acsm1*, *Mogat2*	Y	ureteric trunk, weak	N
Nr2f1/2 (COUP-TF)	Nuclear Receptor	Core, ECR	16/25	0-0.004	*Spp2*, *Slc27a2*, *Prodh2*, *Slc18a1*, *Glyctk*, *Slc6a20b*, *AI317395*, *Tcn2*, *Ace*, *Fbp1*, *Slc3a1*, *C2*, *Ugt2b37*, *Acsm1*, *Aqp11*, *Mogat2*	N/A	Nr2f1 in RI, MM, SSB, podocyte but not PT. Nr2f2, in all MM and RI but not UB	Y/N
Nr4a2	Nuclear Receptor	Core	16/25	0-0.024	*Slc18a1*, *Glyctk*, *Slc6a20b*, *Mettl7b*, *AI317395*, *BC089597*, *Tcn2*, *Ace*, *Cryl1*, *C2*, *Slc22a6*, *Slc27a2*, *Ugt2b37*, *Cml1*, *Acsm1*, *Aqp11*	N/A	early tubule	Y
Hnf1a	Helix-Turn-Helix	Core, ECR	12/25	0-0.042	*Prodh2*, *Glyctk*, *Mettl7b*, *Fbp1*, *Cryl1*, *Slc3a*, *Slc22a61*, *Slc27a2*, *Ugt2b37*, *Cml1*, *Acsm1*, *Aqp11*	Y	MM, RV, MI, SSB, PT	Y
Hnf1b	Helix-Turn-Helix	Core, ECR	12/25	0-0.015	*Spp2*, *Prodh2*, *Glyctk*,*Tcn2*, *Entpd5*, *Fbp1*, *Cryl1*, *Slc22a6*, *Slc27a2*, *Cml1*, *Acsm1*, *Aqp11*	Y	RV, SSB, PT, LOH, DT, UT	Y
Rbpj	bHLH	Core	11/25	0.023-0.044	*Fmo2*, *Slc27a2*, *Sord*, *Ugt2b37*, *Acsm1*, *Aqp11*, *Slc6a20b*, *Fbp1*, *Cryl1*, *C2*, *Slc22a6*	Y	CM, early tubule	Y
Ace2	Zinc-coordinating	ECR	10/13	0-0.006	*Fmo2*, *Spp2*, *Slc27a2*, *Prodh2*, *Slc6a20b*, *Ace*, *C2*, *Slc18a1*, *Glytck*, *Mettl7b*	Y	PT, SSB, LOH, RI	Y
Snai	Zinc-coordinating	ECR	7/13	0.001-0.021	*Spp2*, *Slc27a2*, *C2*, *Fbp1*, *Ace*, *Prodh2*, *Slc18a1*	Y	RI/vasc	N
Phd1/Egln2	N/A	ECR	8/13	0.01	*Spp2*, *Slc27a2*,*Ugt2b37*, *Aqp11*, *C2*, *Slc18a1*, *Glytck*, *Mettl7b*	N/A	MM; CM/RV around birth	N

CLOVER analysis report of transcription factor binding site (TFBS) motif from JASPAR motif library (TF Motif), transcription factor family (TF class), anchor gene promoter sequence analysis used to predict TF motif (Promoter seq. set) Core  =  Mouse RefSeq gene promoter sequences at -500/+200bp from transcription start site; ECR  =  evolutionarily conserved regions within RefSeq promoter sequence. Count and list of individual anchor genes (# genes and Target Genes respectively) with predicted TFBS motifs based on p-value (p-value). Published observations of renal defects based on transcription factor genetic manipulation studies (Renal defect), GUDMAP-based ISH kidney expression annotation (Expression in developing kidney), and GUDMAP and/or literature evidence for expression/association with early proximal tubule (EPT expression). RI  =  renal interstitium (including medullary (MI) and cortical interstitial (CI) compartments); MM  =  metanephric mesenchyme; CM  =  cap mesenchyme; RV  =  renal vesicle; SSB  =  S-shaped body; PT  =  proximal tubule; LOH  =  loop of Henle; DT  =  distal tubule; vasc  =  vasculature; UT  =  ureteric tip; UB  =  ureteric bud. Early tubule may include RV and/or SSB stage nephrons.

In addition to *Hnf4a*, this analysis also detected other nuclear receptor superfamily TF motifs including sites for binding of the *PPARγ*:*RxRA* heterodimer activation complex, *COUP-TF*I, and *Nr4a2*. Activation of perioxisome proliferator-activated receptor gamma (*PPARγ*) requires heterodimerization of *RxRA* and is therefore represented as *PPARγ*:*RxRA.* The presence of TF motifs may reflect a role for such proteins in proximal tubular gene expression or in the regulation of these genes in another context. *B*oth *PPARγ* and *RxRA* expression have been detected in the proximal tubule [50] and are involved in modulating sodium transport [51]. Although previously only associated with nervous system development, our own data shows that *Nr4a2* is expressed in early nephron tubules (GUDMAP:5209), suggesting a role in the regulation of later proximal tubule specification. *Nr2f1* (COUP-TFI) expression has only been detected in the interstitial mesenchyme (GUDMAP:5232), however it is possible that this transcription factor plays a role in the repression of expression of these key proximal tubular markers. Alternatively, as the JASPAR motif library used does not include all COUP-TF motifs, it is possible that the predicted COUP-TFI sites actually represent regulation by another member of the family. *Nr2f2* (COUP-TFII) expression was strongest in cap mesenchyme, persisted in transitioning nephron structures, but was absent in the adult proximal tubule [50] making this unlikely to be activating EPT anchor gene expression. Tissue-specific transcription factors are thought to play a crucial role by regulating specific transcription networks and signaling pathways. The identification of *RbpJ* confirms the involvement of the Notch pathway in specification of EPT fate [14], [52]. A recent study has also shown both *Hnf1a* and *Hnf1b* act upstream of the Notch pathway to specify epithelial cell fate and differentiation in the gut [53]. A similar pathway could exist in proximal tubule epithelial development.

ECR-based promoter analysis also identified conserved motifs for *Ace2*, *Snai* proteins and *Phd1* within the EPT anchor genes. *Ace2* expression has been previously described within the proximal tubule [5]. However, its known role in this structure is not as a transcription factor but as a collectrin-like cofactor that assists in the delivery of amino acid transporters to the apical surface of the proximal tubular epithelium. As a result, disruption to *Ace2* can result in defects in amino acid absorption including Hartnup disease [5], diabetic injury, and glomerulosclerosis [54]. Evidence for direct transcriptional regulation of the expression of these same amino acid transporters is a novel finding. *Snai* transcription factors are zinc finger transcription factors classically associated with inducing and maintaining a mesenchymal phenotype [55], [56]). Within 15.5dpc kidney, we show that *Snai1* is primarily detected in the cortical interstitium (GUDMAP:13622). While its role in development remains elusive, *Snai1* has been extensively documented in renal fibrosis through the EMT pathway [57]. Hence, the presence of *Snai* motifs within EPT anchor gene promoters would imply that *Snai* suppression of these genes is critical for EMT.

**Figure 6:**
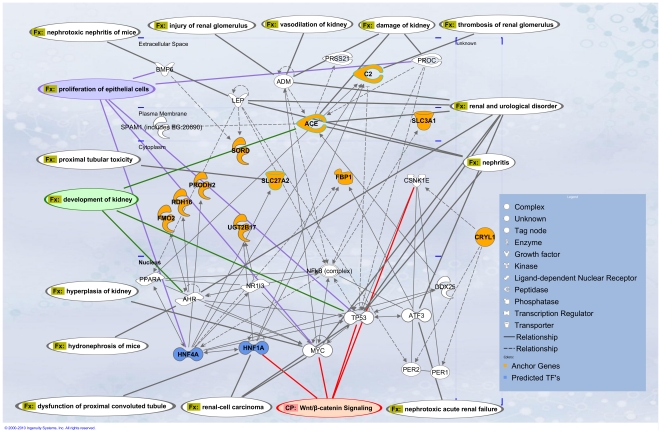
Ingenuity/GO analyses of relationships between proximal tubule anchor genes. Functional network analysis of early proximal tubule anchor genes. Network contains gene/gene products represented as nodes and biological relationship between the nodes as edges as curated in the Ingenuity Knowledge Base. Anchor gene nodes (orange) and transcription factors predicted from TF binding site motif analysis (blue) are highlighted. Kidney related functions and disease are represented in white text boxes. Functions directly related to proximal tubule development are outlined in purple, green and red.

To identify other potential tissue-specific functional networks of proximal tubule, the 25 EPT anchor genes were used for genetic network analysis using Ingenuity Pathway Analysis. Network interactions were inferred using the Ingenuity Knowledge Base of pre-curated molecular and functional interactions mined from published literature. The networks are displayed as nodes (genes) and edges (biological relationships). Networks from 11 genes were merged as one network (Figure 6). This then highlights the direct relationship of genes required for epithelial differentiation to main proximal tubule identity and also proximal tubule function. Many of the genes are also implicated in renal disease, suggesting that a loss of precise regulation of tissue specific genes is detrimental. The analysis also re-identified involvement of several candidate transcription factors, including the previously identified *Hnf4a*, *Hnf1a* and members of the *PPAR* transcription factor family. Other transcription factors identified in this network included *Ahr* and *p53*, both of which are known to play a role in kidney development [58], [59]. These networks and pathways highlight the potential importance of maintaining proximal tubular identity where perturbations to these nodes can lead to diseases.

In summary, this study has defined a stringent bioinformatic approach for the selection of compartment-specific anchor genes and has applied high resolution SISH to the validation and detailed temporospatial characterisation of these genes during murine kidney organogenesis. Reiterative subdivision, profiling and high resolution validation will ultimately facilitate even deeper analysis of kidney development via the generation of tools with which to more accurately select the starting population of cells. Utilizing the anchor genes identified in this study, the generation of anchor-gene driven reporter mice will allow more defined populations to be identified. Such an ability to selectively and specifically target microanatomical compartments of this developing organ will enormously improve our capacity to dissect the genetics of mammalian kidney development by facilitating live imaging, lineage tracing, cellular subfractionation and conditional gene knockout. Ultimately, the information gained from such studies will inform both organogenesis and pathology, potentially leading to approaches that will tackle renal malformations and renal failure.

## Materials and Methods

### Ethics statement

All animal work contributing to this manuscript was conducted according to all state, national and international guidelines. Animal ethics approval was provided by AEEC3 of The University of Queensland (Approval IMB/572/08/NIH (NF)).

### Microarray normalization and analysis

All microarray data discussed in this manuscript was generated and previously described by Brunskill et al (2008) [25]. All data was collected and recorded in a MIAME compliant fashion and is readily accessible via the GUDMAP website (http://www.gudmap.org) as well as via GEO, as detailed previously [25]. Raw. CEL (Affymetrix M430v2) files were obtained for all compartments except early tissues (ureteric bud and metanephric mesenchyme (E11.5)) and pelvic regions (urothelium and uretral smooth muscle from (GEO ID: GSE6290, 6589, 8232, 8360, 12588)) and 11 compartments were chosen for analysis: ureteric tip, cortical collecting duct, medullary collecting duct,, renal vesicle, S-shaped body, renal corpuscle/glomerulus, loop of Henle, proximal tubule, cortical interstitium, medullary interstitium and cap mesenchyme. Normalization of the. CEL files was performed using RMA in Genespring (GX 7.3.1 (Agilent)). Differential expression was defined using a Welch ANOVA test (P <0.01) plus Benjamini and Hochberg correction to define a cohort with dynamic expression across all sub-compartments. All genes were required to have a minimum of 2 fold expression and 100RFU for raw signal. Proximal tubule candidate anchor genes/probesets were further filtered to those with a minimum of 10 fold-change difference to reduce the number of targets for validation.

### Design of *in situ* hybridisation probes/Riboprobe generation tool

Primers were designed to amplify 500–800 bp product primarily towards the 3′UTR region of each transcript using the Primer3 program (http://frodo.wi.mit.edu/). In each case the reverse primer included a T7 polymerase promoter tag for the generation of an antisense riboprobe using T7RNA polymerase. Custom-made tracks of the riboprobe amplicons and primers were generated for visualization on the UCSC genome browser [60] against genomic regions, transcripts, and corresponding Affymetrix probesets. Instructions for track visualization and riboprobe selection are available (http://uqgudmap.imb.uq.edu.au/tools).

For the riboprobe generation tool, all Affymetrix probes were mapped to a non-redundant set of full length mouse cDNA transcripts [61] and RefSeq genes for the mouse and riboprobe amplicons designed so as to generate riboprobes of 500–800 bp. Given the transcriptional complexity of some loci and the dynamic nature of transcript and locus annotations over time, it was important to capture the exact region spanning at least 80% of the 11 Affymetrix probes to minimize the variable expression patterns between the array and ISH. Probe sequences from probesets are matched to the corresponding DNA template from the cDNA and RefSeq set and primers are designed for this region of amplification. Where multiple probe sets existed for a locus, the physical overlap of probe sets with exonic sequences used to generate the riboprobe and a focus towards the 3′ end of transcripts was also prioritized. This pipeline, including all results from the mappings of the non-redundant full length cDNA transcripts and Affymetrix probesets is accessible through our Affymetrix probe design tool (http://uqgudmap.imb.uq.edu.au/riboprobe_design/).

### Tissue collection and processing

Outbred CD1 mouse embryos of unknown sex (both male and female) were collected at 10.5, 13.5 and 15.5 days post coitum (dpc) (Theiler Stages 17, 21, 23) from pregnant adult female mice which were sacrificed by cervical dislocation. The embryonic kidneys and adult kidneys (from either pregnant or non-pregnant females or males) were dissected in ice-cold Phosphate Buffered Saline (PBS) and fixed overnight in fresh 4% paraformaldehyde in PBS at 4°C.

### Section mRNA *in situ* hybridisation

Expression patterns were analysed by RNA section *in situ* hybridisation using digoxigenin (DIG)-labelled antisense riboprobes as described previously [4], [38], [62]. The protocol is also available on the GUDMAP (Genitourinary Development Molecular Anatomy Project) gene expression database (http://www.gudmap.org). Briefly, kidneys were processed and embedded in paraffin and sectioned at 7 µm. Following dewaxing and rehydration, sections were fixed with 4% paraformaldehyde in PBS, washed with PBS, assembled into slide chambers and inserted into the Tecan Freedom Evo 150 robot. Sections were permeabilised with Proteinase K (10 µg/ml) for 20–30minutes and acetylated (0.1 M triethanolamine, 0.65%HCl, 0.25% (v/v) acetic anhydride). Sections were immersed in pre-hybridisation solution whilst the chamber racks were heated from 25°C to 68°C. Hybridisation occurred at 68°C for 10 hours with 0.5 µg/ml of probe in hybridisation buffer (50% formamide, 10% dextran sulphate, 1x Denhardt's, 0.2 mg/ml yeast tRNA, 0.5 mg/ml salmon sperm). After a series of SSC stringency washes, some sections were treated with 2 µg/ml RNase A and all sections were blocked (20% sheep serum, 2% Blocking Reagent in 1x MBST (100 mM Maleic acid, 150 mM NaCl, 0.1% Tween-20, pH7.5) and incubated with 1∶4000 of anti-DIG-alkaline phosphatase Fab fragments for 6 hours at 4°C. Sections were washed with NTMT (0.1 M NaCl, 0.1 M Tris.HCl pH9.6, 50 mM MgCl2, 0.1% Tween20). Chromogenic substrate BM Purple was used to detect the *in situ* alkaline phosphatase activity. Once the signal had reached optimal intensity (4–100 hr), the slides were rinsed and fixed in 4% paraformaldehyde/PBS at 25°C for 10 min followed by PBS washes in order to preserve the mRNA *in situ* hybridisation signal. In order to keep the level of false negatives to a minimum, control riboprobes for monitoring high and low gene expression (*Wnt4* and *Shh* respectively) were included in every hybridisation run. Resulting sections were scanned using a semi-automated. slide System from Olympus and Soft Imaging Systems (BX51 microscope, digital CCD camera, motorized scanning stage and workstation, automated slide loader and. slide software) and images captured using Olyvia software (Soft Imaging Systems, Olympus) and Adobe Photoshop CS2. All analyses presented (both microarray and SISH) are for genes from 3 compartments of the 15.5dpc mouse kidney, including; early proximal tubule (EPT), renal vesicle (RV) and renal medullary interstitium (MI). It should be noted that RV genes originated from microarrays hybridised with renal vesicles obtained from 12.5dpc kidneys by LCM.

### Enrichment for transcription factor binding sites and functional network analysis

Transcription factor binding site (TFBS) motifs was scanned within two sets of core promoter regions from proximal tubule anchor genes; a) the entire core promoter sequence (+500 bp/−200 bp from the transcription start site retrieved from UCSC Genome Browser, and b) only evolutionary conserved sequences within the core promoter set with at least 70% identity conserved across a minimum of three species from the ECR Browser using the CLOVER algorithm [42]. Background sequence sets from mouse Chromosome 19 and mouse CpG islands were used as recommended. Transcription factor matrix library of all species was used to infer a broader set of potential TFBS to compensate for some missing TFBS motifs within the vertebrate collection obtained from JASPAR [43].

Proximal tubule anchor genes network and GO analysis was generated by Ingenuity Pathway Analysis (http://www.ingenuity.com), using default settings and gene interactions curated in the Knowledge Base database. Predicted gene nodes without direct interactions to anchor genes were excluded from the network diagram.

## Supporting Information

Figure S1 **Domains of gene expression in the early proximal tubule at 15.5dpc.** Expression analysis of early proximal tubule (EPT)-specific genes was performed using SISH of 15.5dpc kidneys to validate the genes identified through microarray profiling. **A)** Schematic of capillary loop (Stage III) and maturing nephron (Stage IV) subdivided into renal tubular and renal corpuscle structures. The EPT of maturing nephrons was subdivided into presumptive S1, S2 & S3 segments based on histology and anatomical location within the kidney. **B)** Example SISH images of expression domains seen in EPT of Stage III (a) and Stage IV (b-d) nephrons at 15.5dpc. (a) *Gpd1* in Stage III nephron (EPT and anlage of loop of Henle). The entire nephron can be seen from ureteric tip to renal corpuscle (RC). The outside edge of the kidney and nephron are outlined. Expression in visceral epithelium of RC is indicated (arrowhead). (b) *Gpd1* in EPT (arrows), the proximal portion of immature loop of Henle (ILH, arrowheads) and the adjacent S3 EPT (open arrowhead). *Gpd1* was absent from the distal portion of the ILH within the medulla. (c) *Spp2* was specific to the EPT (S1 & S2). Enlarged region (c′) shows *Spp2* in S1 adjacent to RC. (d) *Gyk* in EPT and ILH. Enlarged region (d′) shows *Gyk* in S3 EPT and ILH.
(TIF)

Figure S2 **Spatial expression analysis of early proximal tubule-specific genes in the adult kidney.** SISH of adult kidney revealed domains of expression in the renal proximal tubule of the mature Stage IV nephron. Transverse sections through the whole kidney (top) with high magnifications of two enlarged regions shown below; S3 in the outer stripe of outer medulla (a-d) and S1, S2 and a small subset of S3 in the renal cortex (e-h). Examples are shown from each of the adult expression types; broad expression in all renal proximal tubules S1, S2 and S3 (*Fbp1* – note expression was absent from the first portion of S1 adjacent to the renal corpuscle but present in other S1, S2 and S3) and regional expression in either proximal convoluted tubule (*Acaa1b* and *Spp2* - S2 and a subset of S1); or proximal straight tubule (*Slc3a1* - S3).
(TIF)

Table S1 **Complete list of all genes selected for SISH validation.** All candidate anchor genes identified in microarray analysis from both 11 and 7 kidney-subcompartments. “Array Information” contains Affy 430.2 probeset IDs (Affy ID), probese raw signal intensity in raw florescent units (Raw), fold-change values based on microarray normalization performed in GeneSpring software, median raw signal intensity across other subcompartments (raw signal/normalized). “SISH expression pattern across the entire TS23 kidney” contains gene expression pattern annotations scored against all known 15.5 kidney subcompartments. Candidate genes without probes were due to unavailable Fantom 2 amplicon/clones (Riken), or failed PCR.
(XLS)
