# Supplementary figures and images for: Identification of Anchor Genes during Kidney Development Defines Ontological Relationships, Molecular Subcompartments and Regulatory Pathways
Source: PLoS One. 2011 Feb 28;6(2):e17286. doi: 10.1371/journal.pone.0017286 (PMC3046260; doi:10.1371/journal.pone.0017286)

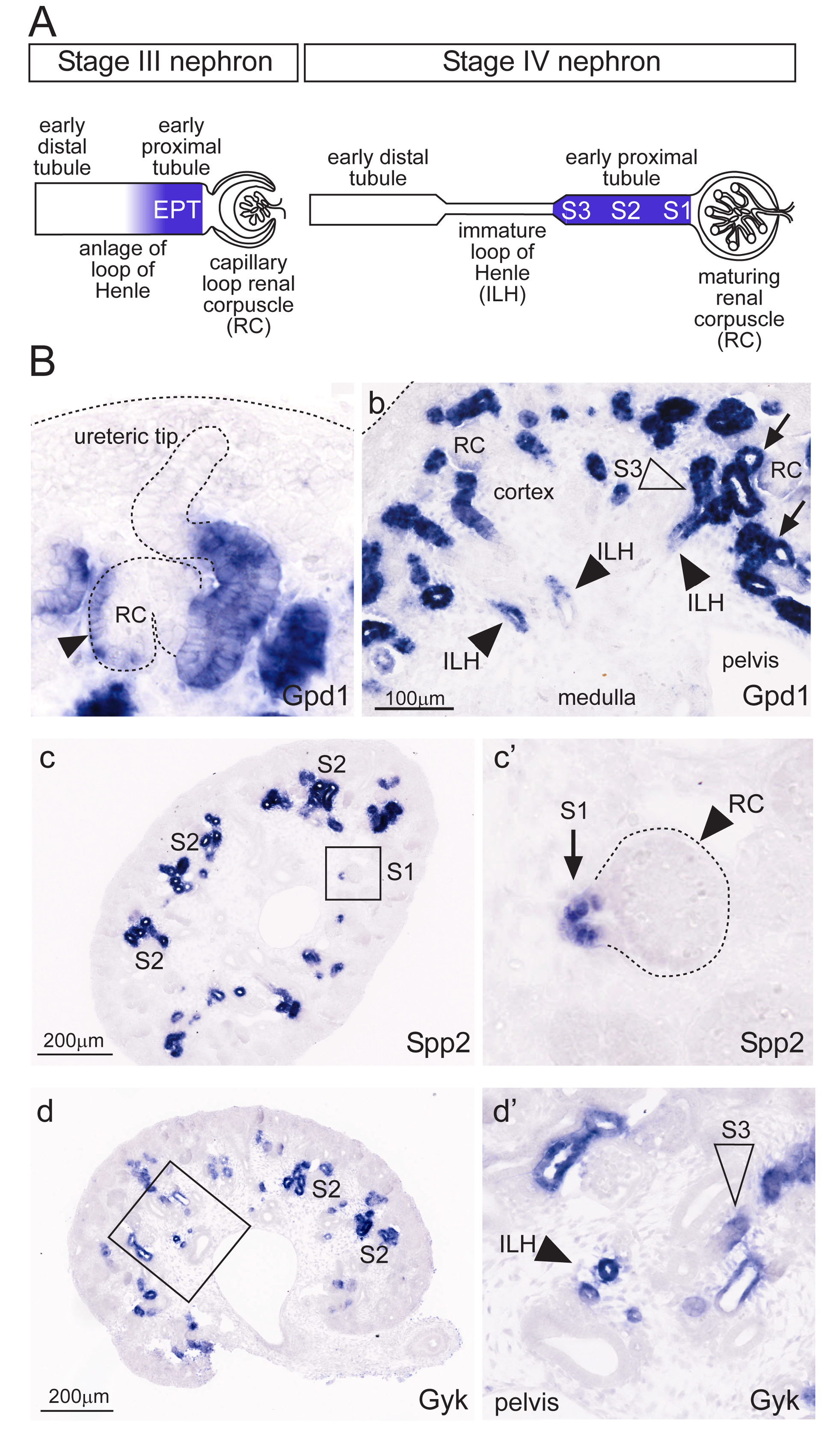

Supplement: Figure S1 — Domains of gene expression in the early proximal tubule at 15.5dpc. Expression analysis of early proximal tubule (EPT)-specific genes was performed using SISH of 15.5dpc kidneys to validate the genes identified through microarray profiling. A) Schematic of capillary loop (Stage III) and maturing nephron (Stage IV) subdivided into renal tubular and renal corpuscle structures. The EPT of maturing nephrons was subdivided into presumptive S1, S2 & S3 segments based on histology and anatomical location within the kidney. B) Example SISH images of expression domains seen in EPT of Stage III (a) and Stage IV (b-d) nephrons at 15.5dpc. (a) Gpd1 in Stage III nephron (EPT and anlage of loop of Henle). The entire nephron can be seen from ureteric tip to renal corpuscle (RC). The outside edge of the kidney and nephron are outlined. Expression in visceral epithelium of RC is indicated (arrowhead). (b) Gpd1 in EPT (arrows), the proximal portion of immature loop of Henle (ILH, arrowheads) and the adjacent S3 EPT (open arrowhead). Gpd1 was absent from the distal portion of the ILH within the medulla. (c) Spp2 was specific to the EPT (S1 & S2). Enlarged region (c′) shows Spp2 in S1 adjacent to RC. (d) Gyk in EPT and ILH. Enlarged region (d′) shows Gyk in S3 EPT and ILH. (TIF) [file pone.0017286.s001.tif]

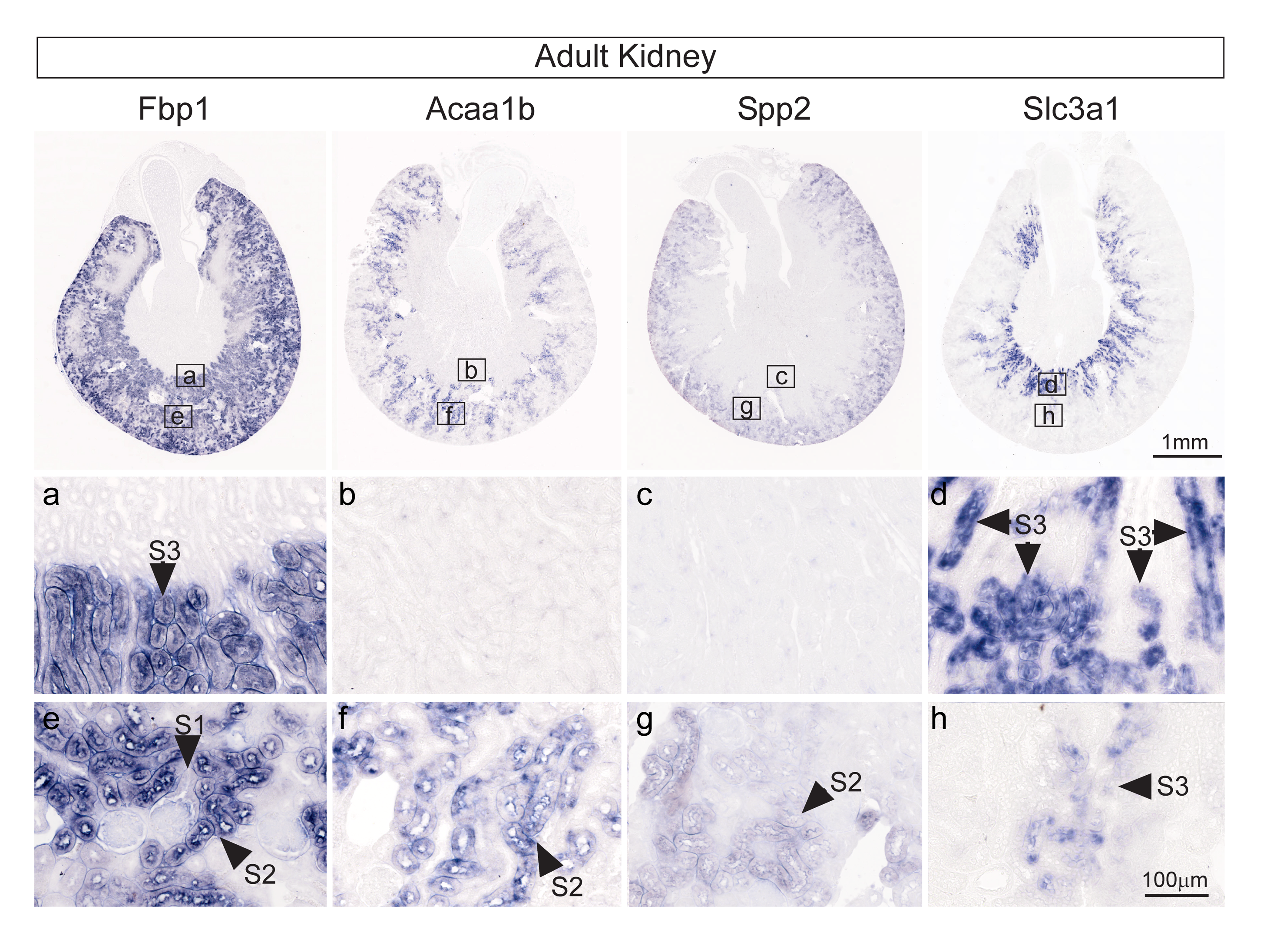

Supplement: Figure S2 — Spatial expression analysis of early proximal tubule-specific genes in the adult kidney. SISH of adult kidney revealed domains of expression in the renal proximal tubule of the mature Stage IV nephron. Transverse sections through the whole kidney (top) with high magnifications of two enlarged regions shown below; S3 in the outer stripe of outer medulla (a-d) and S1, S2 and a small subset of S3 in the renal cortex (e-h). Examples are shown from each of the adult expression types; broad expression in all renal proximal tubules S1, S2 and S3 (Fbp1 – note expression was absent from the first portion of S1 adjacent to the renal corpuscle but present in other S1, S2 and S3) and regional expression in either proximal convoluted tubule (Acaa1b and Spp2 - S2 and a subset of S1); or proximal straight tubule (Slc3a1 - S3). (TIF) [file pone.0017286.s002.tif]
